# Patterns of Postpartum Primary Care Follow-up and Diabetes-Related Care After Diagnosis of Gestational Diabetes

**DOI:** 10.1001/jamanetworkopen.2022.54765

**Published:** 2023-02-06

**Authors:** Rachel D’Amico, Djhenne Dalmacy, Jenifer A. Akinduro, Madison Hyer, Stephen Thung, Shengyi Mao, Naleef Fareed, Seuli Bose-Brill

**Affiliations:** 1 Division of General Internal Medicine, Department of Internal Medicine, The Ohio State University College of Medicine, Columbus; 2 Department of Biomedical Informatics, The Ohio State University, Columbus; 3 Department of Obstetrics and Gynecology, Indiana University, Bloomington; 4 Division of Maternal Fetal Medicine, Department of Obstetrics and Gynecology, The Ohio State University College of Medicine, Columbus

## Abstract

**Importance:**

Gestational diabetes (GD) affects up to 10% of pregnancies and increases lifetime risk of type 2 diabetes 10-fold; postpartum diabetes evaluation and primary care follow-up are critical in preventing and detecting type 2 diabetes. Despite clinical guidelines recommending universal follow-up, little remains known about how often individuals with GD access primary care and type 2 diabetes screening.

**Objective:**

To describe patterns of primary care follow-up and diabetes-related care among individuals with and without GD in the first year post partum.

**Design, Setting, and Participants:**

This cohort study used a private insurance claims database to compare follow-up in the first year post partum between individuals with GD, type 2 diabetes, and no diabetes diagnosis. Participants included postpartum individuals aged 15 to 51 years who delivered between 2015 and 2018 and had continuous enrollment from 180 days before to 366 days after the delivery date. Data were analyzed September through October 2021 and reanalyzed November 2022.

**Main Outcomes and Measures:**

Primary care follow-up visits and diabetes-related care (blood glucose testing and diabetes-associated visit diagnoses) were determined by evaluation and management, *Current Procedural Terminology*, and *International Classification of Diseases, Ninth Revision* and *International Statistical Classification of Diseases and Related Health Problems, Tenth Revision* codes, respectively.

**Results:**

A total of 280 131 individuals were identified between 2015 and 2018 (mean age: 31 years; 95% CI, 27-34 years); 12 242 (4.4%) had preexisting type 2 diabetes and 18 432 (6.6%) had GD. A total of 50.9% (95% CI, 49.9%-52.0%) of individuals with GD had primary care follow-up, compared with 67.2% (95% CI, 66.2%-68.2%) of individuals with preexisting type 2 diabetes. A total of 36.2% (95% CI, 35.1%-37.4%) of individuals with GD had diabetes-related care compared with 56.9% (95% CI, 55.7%-58.0%) of individuals with preexisting diabetes. Only 36.0% (95% CI, 34.4%-37.6%) of individuals with GD connected with primary care received clinical guideline concordant care with blood glucose testing 12 weeks post partum.

**Conclusions and Relevance:**

In this cohort study of postpartum individuals, individuals with GD had lower rates of primary care and diabetes-related care compared with those with preexisting type 2 diabetes, and only 36% of those with GD received guideline-recommended blood glucose testing in the first 12 weeks post partum. This illustrates a missed opportunity for early intervention in diabetes surveillance and prevention and demonstrates the need to develop a multidisciplinary approach for postpartum follow-up.

## Key Points

**Question:**

How many patients with gestational diabetes (GD) have appropriate primary care follow-up and diabetes-related care post partum?

**Findings:**

In this cohort study of national insurance claims data for 280 131 individuals, only 50.9% of individuals with GD had primary care follow-up, and 36.2% had diabetes-related care. The rates of primary care follow-up and diabetes-related care for individuals with GD were significantly lower than those for individuals with type 2 diabetes.

**Meaning:**

These results suggest that, despite guidelines recommending universal follow-up to help improve diabetes and cardiovascular outcomes, individuals with GD experience significantly low rates of follow-up.

## Teaser

This cohort study of US insurance claims data examines rates of appropriate postpartum primary care follow-up and diabetes-related care among patients who experienced gestational diabetes.

## Introduction

Gestational diabetes (GD), defined as hyperglycemia with onset in pregnancy, is a common pregnancy complication. It affects up to 1 in 10 US pregnancies,^1^ and that prevalence is continuing to rise.^2^ The significant perinatal risks of GD are well established, including increased rates of intrauterine fetal demise, maternal hypertensive disorders, and need for Cesarean delivery.^3^ Long-terms outcomes associated with GD on glucose tolerance are also beginning to be understood; individuals with GD are at a 10-fold increased lifetime risk of developing type 2 diabetes compared with those without GD,^4^ with the greatest relative risk in the first 5 years after GD diagnosis.^5^ Individuals with GD have twice the risk of cardiovascular events over the next 10 years post partum than those without GD.^6^

Transition from obstetrics to primary care for prevention and management is critical to blunting these effects. Adherence to postpartum follow-up with obstetricians varies widely, from 11% to 50% depending on the population,^7^ and completion of postpartum blood glucose testing within obstetrics is low.^8^ Postpartum primary care serves as a critical component of longitudinal follow-up for conditions like GD, and is recommended by both the American College of Obstetricians and Gynecologists (ACOG) and the Society for Maternal Fetal Medicine.^9^ Postpartum primary care linkage has been demonstrated to improve rates of timely type 2 diabetes screening, postpartum follow-up, and diagnoses of prediabetes.^10^ However, only about half of all postpartum patients follow up with a primary care practitioner in the 12 months after delivery. National data on GD primary care follow-up are sparse and limited to those with continuous private insurance enrollment of over 4 years,^11^ but show low rates of blood glucose follow-up.^12^ A 2020 national claims study^13^ showed significant lapses in primary care follow-up postpartum for patients with another common pregnancy complication, hypertensive disorders of pregnancy, suggesting poor primary care linkage for those with pregnancy complications that require longitudinal monitoring.

The American Diabetes Association (ADA) and ACOG both recommend the following postpartum interventions for patients with a history of GD: (1) oral glucose tolerance testing (OGTT) at 4 to 12 weeks post partum, (2) discussion of lifestyle and pharmacologic interventions to prevent diabetes development, and (3) ongoing glucose monitoring every 1 to 3 years if not diagnosed with diabetes.^14,15^ OGTT is the recommended diabetes screening test during pregnancy and in the first 12 weeks post partum, as hemoglobin A_1c_ is less sensitive to the rapid glycemic variations and blood volume changes expected with pregnancy.^16^ Glucose follow-up in the first 12 weeks is critical, because during this period placental hormones are no longer affecting glucose but the body’s response to physiologic stress is still apparent, providing a short window into the state of insulin resistance.^17^ Primary care follow-up helps to promote both lifestyle and pharmacologic interventions, which have been shown to prevent and delay onset of type 2 diabetes.^18^ Primary care can also provide ongoing glucose monitoring after the immediate postpartum period given the continued risk of glucose intolerance.

As early diagnosis of type 2 diabetes can decrease long-term diabetes-related morbidity and mortality,^19^ it is critical to longitudinally follow postpartum individuals with GD to ensure prompt detection of diabetes and prediabetes. To gather more data regarding primary care follow-up trends in postpartum GD care, we conducted a retrospective cohort study focusing on primary care follow-up and diabetes-related care.

## Methods

This analysis used a deidentified data set and was deemed exempt for human subject research review by the Office of Responsible Research Practices at the Ohio State University. The data were extracted in September 2021. This study conforms to the Strengthening the Reporting of Observational Studies in Epidemiology (STROBE) reporting guidelines.

### Data Source

Data on women aged 15 to 51 years old who gave birth between 2015 and 2018 were identified from MarketScan (IBM Watson Health), a large administrative database for health care research that contains deidentified data from patients with commercial health insurance or Medicare supplemental health insurance, including inpatient, outpatient, and prescription drug claims.^20,21,22^ Because this database is compliant with the Health Insurance Portability and Accountability Act and contains secondary data, no informed consent was feasible or applicable. Women up to age 51 years, the average age of menopause in the US,^23^ were included given the increase in pregnancy rates in women in their 40s^24^ and the increased risk of GD in women of advanced maternal age.^25^ Consistent with previously published studies,^22,26,27^ eligible patients were identified using claim records (or encounters) containing an *International Classification of Diseases, Ninth Revision (ICD-9)*, *International Statistical Classification of Diseases and Related Health Problems, Tenth Revision (ICD-10)*, or *Current Procedural Terminology* (*CPT*) code indicating the delivery of an infant (eTable 1 in Supplement 1). Using the date of the earliest claim following delivery, patients included in the study had continuous enrollment from 180 days before to 365 days after the delivery date to ensure adequate capture of follow-up. Those with a diagnosis of GD postdelivery were excluded to ensure that hyperglycemia was documented in pregnancy and to avoid incorrect classification of those with ongoing glucose intolerance post partum. Encounters indicating a death in-hospital, missing discharge status, or unknown region were excluded. In instances where a patient had more than 1 record with a delivery code during the study period, only the first entry was analyzed.

### Study Variables

The primary outcome examined were rates of primary care follow-up, which was identified based on postdelivery outpatient encounters that were billed with evaluation and management or preventive care codes (eTable 1 in Supplement 1). Secondary outcomes included follow-up with an indication of diabetes-related care (by either blood glucose testing procedure codes or a diagnosis code related to diabetes or prediabetes) or diabetes-related care encounters among those who had a primary care follow-up. Of note, follow-up not identified as primary care or indicating an infant attached were excluded. To determine the proportion of patients with GD receiving guideline-concordant care of blood glucose testing within 12 weeks post partum,^14^ blood glucose testing (by procedure codes) in the first 12 weeks was considered a secondary outcome. Diabetes diagnosis was the primary independent variable. Patients were classified in 1 of 3 categories based on their diagnosis status (no diabetes, type 2 diabetes, or GD) using *ICD-9* or *ICD-10* codes. Other variables included in the study were age, region, and year of delivery for descriptive purposes. Racial or socioeconomic disparities beyond rurality were not included as variables given the deidentified nature of the database. Rurality was analyzed to test the hypothesis that individuals in what the US Centers for Medicare & Medicaid Services refers to as “super rural areas” (ie, patients residing in zip codes not classified as a metropolitan statistical area) would have lower rates of follow-up and blood glucose testing.^28^ To determine if pregnancy comorbidities affected follow-up rates in patients with GD, hypertensive disorders of pregnancy and preterm birth were analyzed.

### Statistical Analysis

Bivariate association between each outcome and the independent variables was assessed using either a χ^2^ test, 2-sample *t* test, or a Wilcoxon rank-sum test. A confidence interval was reported for each point estimate. A multivariable log-binomial regression model was used to assess the relative risk (RR) of having a primary care follow-up among those who had at least 1 outpatient follow-up relative to diabetes diagnosis status adjusted for age, region, and year of delivery. The model was repeated for each secondary outcome among women who had 1 or more primary follow-up visits, as well as sensitivity analyses for hypertensive disorders of pregnancy and preterm birth. All estimates derived from multivariable log-binomial regression models utilized a maximum likelihood model estimation and the Kenward-Roger method for computing denominator degrees of freedom. The level of statistical significance for all tests was set at α = .05. All statistical analyses were performed using Statistical Analysis System (SAS) software, version 9.4 (SAS Institute).

## Results

A total of 280 131 female participants were identified from 2015 to 2018, with a mean age of 31 years (95% CI, 27-34 years) (Table 1). A total of 249 457 patients (89.1%) had no diagnosis of diabetes, 12 242 patients (4.4%) had a diagnosis of type 2 diabetes, and 18 432 patients (6.6%) had a diagnosis of GD. Regarding year of delivery, 167 580 deliveries (59.8%) were from 2015 to 2016, and 112 551 (40.2%) from 2017 to 2018. The sample involved patients from all US geographic regions, with the South region being the most prevalent (124 233 [44.3%]).

**Table 1.** Characteristics of Sample and Proportions of Primary Care Follow-up

Characteristic	Overall, No. (%)	No primary care follow-up, No. (%) [95% CI]	Primary care follow-up, No. (%) [95% CI]
No. (%)	280 131	181 540 (64.8)	98 591 (35.2)
Diabetes diagnosis			
No diabetes	249 457 (89.1)	168 487 (67.5) [67.3-67.8]	80 970 (32.5) [32.1-32.8]
Type 2 diabetes	12 242 (4.4)	4012 (32.8) [31.3-34.2]	8230 (67.2) [66.2-68.2]
GD	18 432 (6.6)	9041 (49.1) [48.0-50.1]	9391 (50.9) [49.9-52.0]
Age, mean (95% CI), y	31 (27-34)	31 (27-34)	32 (28-35)
Delivery year			
2015-2016	167 580 (59.8)	109 113 (65.1) [64.8-65.4]	58 467 (34.9) [34.5-35.3]
2017-2018	112 551 (40.2)	72 427 (64.4) [64.0-64.7]	40 124 (35.6) [35.2-36.1]
Region			
North Central	60 694 (21.7)	40 317 (66.4) [66.0-66.9]	20 377 (33.6) [32.9-34.2]
Northeast	48 729 (17.4)	29 787 (61.1) [60.6-61.7]	18 942 (38.9) [38.2-39.6]
South	124 233 (44.3)	82 294 (66.2) [65.9-66.6]	41 939 (33.8) [33.3-34.2]
West	46 475 (16.6)	29 142 (62.7) [62.1-63.3]	17 333 (37.3) [36.6-38.0]
Super rural	28 799 (10.3)	19 738 (68.5) [67.9-69.2]	9061 (31.5) [30.5-32.4]

Abbreviation: GD, gestational diabetes.

### Primary Care Follow-up

Overall, 98 591 patients (35.2%) accessed primary care in the first 12 months post partum (Table 1). Among patients with no diabetes diagnosis, only 32.5% (95% CI, 32.1%-32.8%) had primary care follow-up in the first year post partum. Of individuals with GD, 50.9% (95% CI, 49.9%-52.0%) received primary care follow-up, whereas 67.2% (95% CI, 66.2%-68.2%) of individuals with type 2 diabetes received primary care follow-up in the year post partum. Women with GD had a RR for accessing primary care of 0.78 (95% CI, 0.76-0.79) compared with those with type 2 diabetes. Patients in the Northeast region had the highest rates of follow-up (38.9%; 95% CI, 38.2%-39.6%). Patients living in super rural regions had lower rates of follow-up than the general sample, with 31.5% (95% CI, 30.5%-32.4%) accessing primary care in the first year post partum.

### Diabetes-Related Care Trends

Overall, 42 711 patients (15.2%) received diabetes-related care (Table 2). Of patients with GD, 36.2% (95% CI, 35.1%-37.4%) had diabetes-related care, compared with 56.9% of patients with type 2 diabetes (95% CI, 55.7%-58.0%). Of patients with no diabetes diagnosis, 11.7% received diabetes-related care (95% CI, 11.3%-12.0%). The most common type of laboratory values for blood glucose followup were hemoglobin A_1c_ followed by glucose measurement.

**Table 2.** Characteristics of Sample and Proportions of Diabetes-Related Care

Characteristic	Overall, No. (%)	No diabetes-related care, No. (%) [95% CI]	Diabetes-related care, No. (%) [95% CI]
No. (%)	280 131	237 420 (84.8)	42 711 (15.2)
Diabetes diagnosis			
No diabetes	249 457 (89.1)	220 382 (88.3) [88.2-88.5]	29 0975 (11.7) [11.3-12.0]
Type 2 diabetes	12 242 (4.4)	5279 (43.1) [41.8-44.5]	6963 (56.9) [55.7-58.0]
GD	18 432 (6.6)	11 759 (63.8) [62.9-64.7]	6673 (36.2) [35.1-37.4]
Age, mean (95% CI), y	31 (27-34)	31 (27-34)	32 (29-36)
Year of delivery			
2015-2016	167 580 (59.8)	143 100 (85.4) [85.2-85.6]	24 480 (14.6) [14.2-15.1]
2017-2018	112 551 (40.2)	94 430 (83.8) [83.6-84.0]	18 231 (16.2) [15.7-16.7]
Region			
North Central	60 694 (21.7)	52 259 (86.1) [85.8-86.4]	8435 (13.9) [13.2-14.6]
Northeast	48 729 (17.4)	39 768 (81.6) [81.2-82.0]	8961 (18.4) [17.6-19.2]
South	124 233 (44.3)	105 928 (85.3) [85.1-85.5]	18 305 (14.7) [14.2-15.2]
West	46 475 (16.6)	39 465 (84.9) [84.6-85.3]	7010 (15.1) [14.2-15.9]
Super rural	28 799 (10.3)	25 550 (88.7) [88.3-89.1]	3249 (11.3) [10.2-12.4]

Abbreviation: GD, gestational diabetes.

The rate of diabetes-related care increased from 14.6% (95% CI, 14.2%-15.1%) in 2015-2016 to 16.2% (95% CI, 15.7%-16.7%) in 2017-2018. Patients living in the Northeast region had the highest rates of glucose testing (18.4%, 95% CI, 17.6%-19.2%). Patients living in super rural regions had lower rates of diabetes-related care than the overall sample; only 11.3% (95% CI 10.2%-12.4%) had diabetes-related care in the first year post partum.

### Diabetes-Related Care Among Those With Primary Care Follow-up

Not only were the rates of follow-up in primary care low, but the rates of diabetes-related care for those connected with primary care were low (Table 3). Of patients with GD who accessed primary care, only 71.1% (95% CI, 70.0%-72.1%) received diabetes-related care in the first 12 months post partum. Of patients with type 2 diabetes who accessed primary care, 84.6% (95% CI, 83.8%-85.5%) received diabetes-related care. Patients established in primary care without follow-up blood glucose testing were more likely to have had fewer total follow-up visits (Table 3). Among those established in primary care, women with GD had a RR of 0.85 (95% CI, 0.83-0.86) of receiving diabetes-related care compared with those with type 2 diabetes (Table 4).

**Table 3.** Characteristics of Sample and Proportions of Diabetes-Related Care Among Primary Care Follow-up

Characteristic	Overall, No. (%)	No diabetes-related care, No. (%) [95% CI]	Diabetes-related care, No. (%) [95% CI]
No. (%)	98 591	55 880 (56.7)	42 711 (43.3)
Diabetes diagnosis			
No diabetes	80 970 (82.1)	51 895 (64.1) [63.7-64.5]	29 075 (35.9) [35.4-36.5]
Type 2 diabetes	8230 (8.3)	1267 (15.4) [13.4-17.4]	6963 (84.6) [83.8-85.5]
GD	9391 (9.5)	2718 (28.9) [27.2-30.6]	6673 (71.1) [70.0-72.1]
Age, mean (95% CI), y	32 (28-35)	31 (27-34)	32 (29-36)
Time to first follow-up, median (IQR), d	31 (8-67)	36 (10-97)	25 (6-46)
Preventive care	8049 (8.2)	6969 (86.6) [85.8-87.4]	1080 (13.4) [11.4-15.5]
Evaluation and management code	57 555 (58.4)	48 911 (85.0) [84.7-85.3]	8644 (15.0) [14.3-15.8]
No. of follow-up visits			
1	6941 (7.0)	6301 (90.8) [90.1-91.5]	640 (9.2) [7.0-11.5]
2	8448 (8.6)	6572 (77.8) [76.8-78.8]	1876 (22.2) [20.3-24.1]
3	9086 (9.2)	6402 (70.5) [69.3-71.6]	2684 (29.5) [27.8-31.3]
4	8930 (9.1)	5716 (64.0) [62.8-65.3]	3214 (36.0) [34.3-37.7]
≥5	65 186 (66.1)	30 889 (47.4) [46.8-47.9]	34 297 (52.6) [52.1-53.1]
Year of delivery			
2015-2016	58 467 (59.3)	33 987 (58.1) [57.6-58.7]	24 480 (41.9) [41.3-42.5]
2017-2018	40 124 (40.7)	21 893 (54.6) [53.9-55.2]	18 231 (45.4) [44.7-46.2]
Region			
North Central	20 377 (20.7)	11 942 (57.6) [57.7-59.5]	8435 (41.4) [40.3-42.4]
Northeast	18 942 (19.2)	9981 (52.7) [51.7-53.7]	8961 (47.3) [46.3-48.3]
South	41 939 (42.5)	23 634 (56.4) [55.7-57.0]	18 305 (43.6) [42.9-44.4]
West	17 333 (17.6)	10 323 (59.6) [58.6-60.5]	7010 (40.4) [39.3-41.6]
Super rural	9061 (9.2)	5812 (64.1) [62.9-65.4]	3249 (35.9) [34.2-37.5]

Abbreviation: GD, gestational diabetes.

**Table 4.** Adjusted Relative Risk Among Women With a Diabetes or Gestational Diabetes Diagnosis Who Had at Least 1 Outpatient Follow-up

Outcomes	Adjusted relative risk (95% CI)		
	Gestational diabetes vs type II diabetes	Hypertensive disorders of pregnancy vs no HDP	Preterm vs non-preterm
Primary care follow-up	0.78 (0.76-0.79)	1.07 (1.02-1.12)	1.00 (0.95-1.06)
Diabetes-related care^a^	0.85 (0.83-0.86)	0.98 (0.94-1.03)	1.03 (0.98-1.08)
Blood glucose test^a^	0.82 (0.79-0.85)	0.91 (0.82-1.01)	0.95 (0.86-1.06)

Abbreviation: HDP, Hypertensive disorders of pregnancy.

^a^ Among those who had a primary care follow-up.

### Blood Glucose Testing in the First 12 Weeks

Data from the first 12 weeks was examined to determine how closely ADA guidelines for blood glucose testing in the first 12 weeks after delivery^14^ were followed for those established in primary care. Overall, 12.1% of all patients received blood glucose testing in the first 12 weeks (eTable 2 in Supplement 1). In the first 12 weeks post partum, 36.0% (95% CI, 34.4%-37.6%) of those with GD received blood glucose testing. Importantly, 4.8% of all patients received blood glucose via hemoglobin A_1c_ in the first 12 weeks, which is not an appropriate test immediately post partum given high red blood cell turnover and significant short-term glycemic changes. Only 2.0% of patients received an oral glucose tolerance test, the criterion standard test in the immediate postpartum period; 5.5% of patients received serum glucose measurement.

There was an increase in blood glucose testing over time; 11.5% (95% CI, 10.7%-12.2%) of patients seen between 2015 and 2016 had blood glucose testing in the first 12 weeks, and 13.1% (95% CI, 12.2%-14.1%) of patients seen between 2017 and 2018 had blood glucose testing in the first 12 weeks. Rates of blood glucose testing in the first 12 weeks were similar throughout regions. Patients in super rural areas had lower rates of blood glucose testing in the first 12 weeks, with only 9.4% (95% CI, 7.5%-11.4%) receiving blood glucose testing in that time.

### Outcomes Associated With Comorbidities on Primary Care Follow-up

The cohorts were then analyzed by rates of comorbidities. A total of 17 460 women (7.0%) of women with no diabetes diagnosis, 2026 (11.0%) with GD, and 1824 (14.9%) diagnosed with type 2 diabetes had hypertensive disorders of pregnancy (HDP). A total of 14 008 women (5.6%) with no diabetes diagnosis, 1333 (7.23%) with GD, and 1097 (9.0%) diagnosed with type 2 diabetes had preterm birth (Figure). Of women with GD, those with HDP were more likely to have primary care follow-up (OR, 1.16; 95% CI 1.05-1.30); otherwise, there were no statistically significant differences in follow-up or blood glucose testing by pregnancy complication (Table 4). A total 28 131 women (11.3%) with no diabetes diagnosis, 2852 (15.5%) with GD, and 2412 (19.7%) with type 2 diabetes had encounters for hypertension in the first 12 months post partum.

**Figure.**
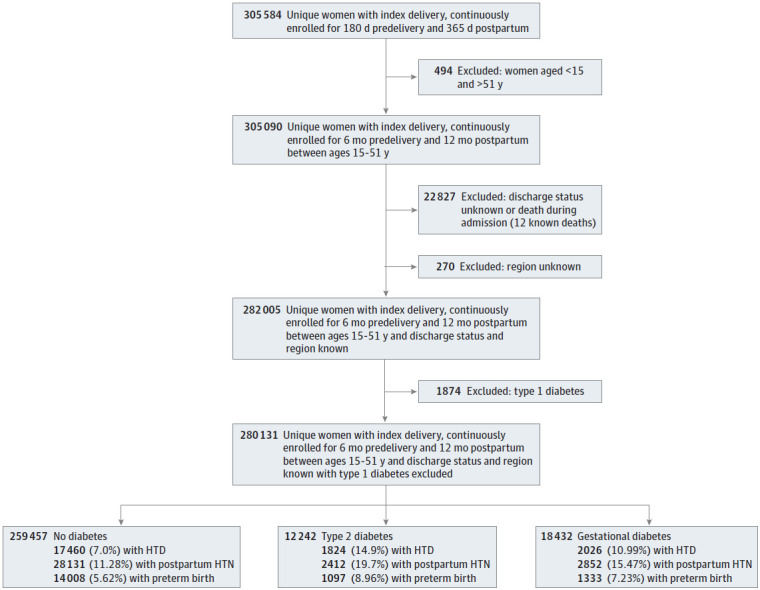
Flow Diagram of Participant Selection HTD indicates hypertensive disorders of pregnancy; HTN, hypertension.

## Discussion

Our data show that individuals with GD throughout the US have low rates of primary care and diabetes-related care. This is one of the first national studies to study primary care follow-up in patients with GD, a key component in the longitudinal follow-up of patients at significantly increased type 2 diabetes risk. While their rates of follow-up are higher than those with no diabetes diagnosis, they are lower than those with preexisting type 2 diabetes and significantly below the recommendation for universal follow-up. Overall, patients with GD had a RR of 0.78 for receiving primary care compared with those with preexisting diabetes in the first year post partum. Even once connected with primary care, there were low rates of diabetes-related care for patients with GD. There were also disparities in postpartum diabetes-related follow-up for patients living in super rural areas; to our knowledge, this is the first study to document rural disparities in postpartum diabetes follow-up in the US. Early follow-up of GD may help to prevent or detect type 2 diabetes earlier, allow opportunities for promotion of improving cardiovascular health, and prevent the significant morbidity related to uncontrolled diabetes. As GD prevalence increases, it is critical we determine how to increase follow-up rates.

Another important and novel element of our data is that, of patients with GD who did receive blood glucose testing, the majority received hemoglobin A_1c_ in the first 12 weeks post partum, which is not an appropriate test in the immediate postpartum period. This may lead to patients with continued insulin resistance being missed due to the significant short-term glycemic changes as well as blood turnover associated with delivery. This suggests a lack of understanding on appropriate follow-up of pregnancy complications for primary care physicians, as hemoglobin A_1c_ is a common diabetes screen in other clinical settings. These rates may be remedied by raising awareness among primary care practitioners about the appropriate follow-up of GD.

Postpartum follow-up has multiple challenges. It is often unclear which clinician, obstetrics or primary care, should be responsible for ensuring follow-up of pregnancy complications like GD. Primary care physicians may be less familiar with recommendations for surveillance of GD, especially because oral glucose tolerance tests are not widely used in other clinical situations. Primary care physicians may not practice in environments that are typically equipped to take care of postpartum individuals, for example lacking connections with lactation counseling or supplemental Women, Infant, Children’s programs. However, primary care physicians are uniquely suited to provide longitudinal care for patients outside of pregnancy and are often familiar with chronic care models that can provide systematic support. About 50% of women with GD go on to develop type 2 diabetes^1^ and primary care physicians have significant expertise in prevention and management of prediabetes and type 2 diabetes, making them a key resource in the postpartum period for patients with GD.

There are promising care models to help improve GD follow-up. Maternal-infant dyad clinics—with a joint encounter for parent and infant—have been shown to increase GD follow-up and appropriate screening.^10^ By taking advantage of other encounters in which parents interface with the health care system, like well child visits, clinicians may be able to connect postpartum patients with the medical care they need. Patient contact with a health educator, via phone or mail, has also been shown to increase follow-up blood glucose testing.^29^ Given that GD is known to be associated with future cardiovascular health, it is important that primary care physicians make follow-up of GD a priority just like other chronic health conditions.

### Limitations

There were several limitations to this study. As this is based on insurance claims of patients with continuous private insurance enrollment, the follow-up rates of patients covered by public insurance (eg, Medicaid) or who are uninsured are not known. Our data would also not capture any blood glucose testing or follow-up that was not billed for or coded incorrectly. This study cannot comment on racial or socioeconomic disparities beyond rurality given the deidentified nature of the database, limiting the understanding of other inequities in follow-up rates. Further studies are needed to characterize follow-up in these patient populations. Previous data have shown worsened glycemic control among pregnant people with higher social vulnerability,^30^ raising concern for potential inequity. Given this study’s focus on characterizing primary care follow-up, follow-up obtained through postpartum checkups were not assessed fully in this study.

## Conclusions

This cohort study demonstrated concerningly low rates of postpartum engagement in what is, to our knowledge, the largest study of primary care follow-up in GD to date. A paradigm shift in how we view GD, not as an acute issue that resolves with birth but as a chronic disease process and independent cardiovascular risk factor, is necessary to ensure appropriate care of patients with GD. Further research is needed to characterize which patients are at the highest risk for not receiving appropriate postpartum follow-up, as well as identifying barriers to post-GD primary care transitions, to further target interventions.

## Notes

Supplement 1. **eTable 1.** *ICD* and *CPT* Codes Utilized for Variable Identification **eTable 2.** Characteristics of Sample and Proportions of Blood Glucose Testing Within the First 12 Weeks Among Primary Care Follow-up

Supplement 2.
Data Sharing Statement
