# Supplementary material for: Patterns of Postpartum Primary Care Follow-up and Diabetes-Related Care After Diagnosis of Gestational Diabetes
Source: JAMA Netw Open. 2023 Feb 6;6(2):e2254765. doi: 10.1001/jamanetworkopen.2022.54765 (PMC12512597; doi:10.1001/jamanetworkopen.2022.54765)
Supplement: Supplement 1. — eTable 1. ICD and CPT Codes Utilized for Variable Identification eTable 2. Characteristics of Sample and Proportions of Blood Glucose Testing Within the First 12 Weeks Among Primary Care Follow-up [file jamanetwopen-e2254765-s001.pdf]

Supplemental Online Content

D'Amico R, Dalmacy D, Akinduro JA, et al. Patterns of postpartum primary care follow-up and diabetes-related care after diagnosis of gestational diabetes. *JAMA Netw Open.* 2023;6(2):e2254765. doi:10.1001/jamanetworkopen.2022.54765

eTable 1. *ICD* and *CPT* Codes Utilized for Variable Identification

eTable 2. Characteristics of Sample and Proportions of Blood Glucose Testing Within the First 12 Weeks Among Primary Care Follow-up

This supplemental material has been provided by the authors to give readers additional information about their work.

© 2023 D'Amico R et al. *JAMA Network Open.*

eTable 1. *ICD* and *CPT* Codes Utilized for Variable Identification

VARIABLE IDENTIFICATION	CODES	SOURCE
Delivery		
	72, 73, 74, 75.4, Z37, Z38, O80, O300, 10D07Z3, 0W8NXZZ with: 10D07Z4, 10D07Z5,10S07ZZ, 10D07Z6, 10D07Z8, 10D07Z7, 10E0XZZ, 0UL50ZZ, 0UL53ZZ, 0UL54ZZ, 0UL60ZZ, 0UL63ZZ, 0UL64ZZ, 0UL70ZZ, 0UL73ZZ, 0UL74ZZ, 10D00Z0, 10D00Z1, 10D00Z2	ICD
	01960, 01961, 01962, 01963, 01967, 01968, 01969, 59050, 59051, 59400, 59409, 59410, 59412, 59414, 59430, 59510, 59514, 59515, 59525, 59610, 59612, 59614, 59618, 59620, 59622	CPT
Primary care	99384, 99385, 99386, 99394, 99395, 99396	Preventive care
	99201, 99202, 99203, 99204, 99205, 99211, 99212, 99213, 99214, 99215	E&M
Diabetes care	E08X E10X E11X E13X, R73, Z794	ICD
	HbA1C: 83036, 83037<br>Oral GTT: 101200, 82951, 82952<br>Blood glucose: 82947, 82948, 82962	CPT
Diabetes status	Gestational diabetes: 648.8, O24, O24.4, 024.4X, O24.9X, P70.0	ICD
	Type 2 diabetes: 648.01, 648.03, E11, Z79.4, O24.1, O24.3, I738, Z794, E08	ICD
	Type 1 diabetes (exclude): E10, O24.0	ICD
Hypertensive disorders of pregnancy	642.3-642.7, O11, O13, O14, O15	ICD
Preterm labor	644.0- 644.2, 765, Z87.51, O09.211- O09.213, O09.219, O60.00, O60.02, O60.03, O60.10X0 - O60.14X	ICD

© 2023 D'Amico R et al. *JAMA Network Open.*

eTable 2. Characteristics of Sample and Proportions of Blood Glucose Testing Within the First 12 Weeks Among Primary Care Follow-up

	Overall n = 100,084	No Blood Glucose Test n = 86 620 (87.9%)		Blood Glucose Test n = 11 971 (12.1%)	
	n (%)	*n (%)*	*95% CI*	*n (%)*	*95% CI*
Diabetes (DM) Diagnosis					
No DM	80 970 (82.1)	76 042 (93.9)	93.7 – 94.1	4928 (6.1)	5.4 – 6.8
Type 2 DM	8230 (8.3)	4570 (55.5)	54.1 – 57.0	3660 (44.5)	42.9 – 46.1
GDM	9391 (9.5)	6008 (64.0)	62.8 – 65.2	3383 (36.0)	34.4 – 37.6
Age (years), Mean	32	31	28 – 35	33	30 – 36
Days to First Follow-up, *Median (IQR)*	18 (6 – 40)	18 (6 – 40)		18 (5 – 40)	
Type of Test					
HbA1C	4774 (4.8)	0 (0.0)	– –	4774 (100.0)	100.0 – 100.0
Oral GTT	2000 (2.0)	0 (0.0)	– –	2000 (100.0)	100.0 – 100.0
Blood glucose	5197 (5.3)	0 (0.0)	– –	5197 (100.0)	100.0 – 100.0
Preventive Care	2,504 (2.5)	2307 (92.1)	91.0 – 93.2	197 (7.9)	4.1 – 11.6
E & M	45 882 (46.5)	43 829 (95.5)	95.3 – 95.7	2053 (4.5)	3.6 – 5.4
Number of Follow-up					
0	20 585 (20.9)	20 585 (100.0)	100.0 – 100.0	0 (0.0)	– –
1	21 453 (21.8)	19 849 (92.5)	92.2 – 92.9	1604 (7.5)	6.2 – 8.8
2	18 501 (18.8)	16 2120 (87.1)	86.6 – 87.6	2381 (12.9)	11.5 – 14.2
3	13 133 (13.3)	10 904 (83.0)	82.3 – 83.7	2229 (17.0)	15.4 – 18.5
4	428 (8.5)	6743 (80.0)	79.1 – 81.0	1685 (20.0)	18.1 – 21.9
≥ 5	16 491 (16.7)	12 419 (75.3)	74.5 – 76.1	4072 (24.7)	23.4 – 26.0
Year of Delivery					
2015 – 2016	58 467 (59.3)	51 771 (88.5)	88.3 – 88.8	6696 (11.5)	10.7 – 12.2

© 2023 D'Amico R et al. *JAMA Network Open.*

	Overall<br>n = 100,084	No Blood Glucose Test<br>n = 86 620 (87.9%)		Blood Glucose Test<br>n = 11 971 (12.1%)	
	n (%)	*n (%)*	*95% CI*	*n (%)*	*95% CI*
Diabetes (DM) Diagnosis					
2017 – 2018	40 124 (40.7)	34 849 (86.9)	86.5 – 87.2	5275 (13.1)	12.2 – 14.1
Region					
North Central	20 337 (20.7)	17 821 (87.5)	87.0 – 87.9	2556 (12.5)	11.3 – 13.8
Northeast	18 492 (19.2)	16 625 (87.8)	87.3 – 88.3	2317 (12.2)	10.9 – 13.6
South	41 939 (42.5)	37 078 (88.4)	88.1 – 88.7	4861 (11.6)	10.7 – 12.5
West	17 333 (17.6)	15 096 (87.1)	86.6 – 87.6	2237 (12.9)	11.5 – 14.3
Super Rural	9061 (9.2)	8208 (90.6)	90.0 – 91.2	853 (9.4)	7.5 – 11.4

© 2023 D’Amico R et al. *JAMA Network Open.*
