# Supplementary material for: Patterns of Postpartum Primary Care Follow-up and Diabetes-Related Care After Diagnosis of Gestational Diabetes
Source: JAMA Netw Open. 2023 Feb 6;6(2):e2254765. doi: 10.1001/jamanetworkopen.2022.54765 (PMC12512597; doi:10.1001/jamanetworkopen.2022.54765)
Supplement: Supplement 2. — Data Sharing Statement [file jamanetwopen-e2254765-s002.pdf]

Data Sharing Statement

D'Amico. Patterns of Postpartum Primary Care Follow-up and Diabetes-Related Care After Diagnosis of Gestational Diabetes. *JAMA Netw Open*. Published February 06, 2023. doi:10.1001/jamanetworkopen.2022.54765

Data
Data available: No

Additional Information
Explanation for why data not available: Data is available through MarketScan database.
